# Genetic Diversity of Viral Populations Associated with *Ananas* Germplasm and Improvement of Virus Diagnostic Protocols

**DOI:** 10.3390/pathogens11121470

**Published:** 2022-12-05

**Authors:** Adriana E. Larrea-Sarmiento, Alejandro Olmedo-Velarde, Xupeng Wang, Wayne Borth, Ryan Domingo, Tracie K. Matsumoto, Jon Y. Suzuki, Marisa M. Wall, Michael J. Melzer, John Hu

**Affiliations:** 1 Department of Plant and Environmental Protection Sciences, University of Hawaii, Honolulu, HI 96822, USA; 2 United States Department of Agriculture, Agricultural Research Service, Daniel K. Inouye U. S. Pacific Basin Agricultural Research Center, Hilo, HI 96720, USA

**Keywords:** *Ananas* spp., germplasm, ampeloviruses, sadwaviruses, badnaviruses, recombination, RT-PCR

## Abstract

Pineapple (*Ananas comosus* L. [Merr.]) accessions from the U.S. Tropical Plant Genetic Resources and Disease Research (TPGRDR) in Hilo, Hawaii were subjected to RNA-sequencing to study the occurrence of viral populations associated with this vegetatively propagated crop. Analysis of high-throughput sequencing data obtained from 24 germplasm accessions and public domain transcriptome shotgun assembly (TSA) data identified two novel sadwaviruses, putatively named “pineapple secovirus C” (PSV-C) and “pineapple secovirus D” (PSV-D). They shared low amino acid sequence identity (from 34.8 to 41.3%) compared with their homologs in the Pro-pol region of the previously reported PSV-A and PSV-B. The complete genome (7485 bp) corresponding to a previously reported partial sequence of the badnavirus, pineapple bacilliform ER virus (PBERV), was retrieved from one of the datasets. Overall, we discovered a total of 69 viral sequences representing ten members within the *Ampelovirus*, *Sadwavirus*, and *Badnavirus* genera. Genetic diversity and recombination events were found in members of the pineapple mealybug wilt-associated virus (PMWaV) complex as well as PSVs. PMWaV-1, -3, and -6 presented recombination events across the quintuple gene block, while no recombination events were found for PMWaV-2. High recombination frequency of the RNA1 and RNA2 molecules from PSV-A and PSV-B were congruent with the diversity found by phylogenetic analyses. Here, we also report the development and improvement of RT-PCR diagnostic protocols for the specific identification and detection of viruses infecting pineapple based on the diverse viral populations characterized in this study. Given the high occurrence of recombination events, diversity, and discovery of viruses found in *Ananas* germplasm, the reported and validated RT-PCR assays represent an important advance for surveillance of viral infections of pineapple.

## 1. Introduction

Pineapple (*Ananas comosus* L. [Merr.]) is a perennial plant in the family *Bromeliaceae*, native to the South American tropics between Paraguay and Brazil. In 2020, worldwide pineapple production was estimated at 28 million metric tons. The Philippines, Costa Rica, and Brazil were the top producers with 2.7, 2.6, and 2.5 million metric tons, respectively. Most of The United States of America (USA) grown pineapple still comes from Hawaii and Puerto Rico, although there is a small-scale production in Florida and California. The USA pineapple production was estimated at 168 thousand metric tons in 2020 placing it as the sixth leading type of fresh fruit in terms of per capita consumption [1]. Cultivated pineapples are referred to as “clones” because they are vegetatively propagated. Since the crop is propagated by crowns, slips, suckers, or other propagules, these clones can often accumulate more than one virus. This accumulation negatively affects plant growth and production.

Pineapple can be infected by RNA and DNA viruses belonging to different families and genera [2,3,4,5,6]. Of more than seven diseases reported on pineapple, mealybug wilt of pineapple (MWP) is the most important and complex viral disease affecting pineapple production worldwide. Symptoms induced by viral infections and associated with MWP involve: wilting, leaf-tip dryness, leaf redness and/or pinkness, leaf inward curling, stuntedness, and root decay [2,7]. Members of several species within the *Ampelovirus* genus (family *Closteroviridae*) have been directly associated, in conjunction with mealybug feeding, with MWP causing severe damage to pineapple production [7,8]. Five distinct mealybug-transmitted ampeloviruses, named pineapple mealybug wilt-associated virus 1 (PMWaV-1), PMWaV-2, PMWaV-3, PMWaV-5, and the recently reported PMWaV-6, have been detected in pineapple [4,5,6,9]. Members of two *Badnavirus* species (family *Caulimoviridae*), pineapple bacilliform CO virus (PBCOV) and pineapple bacilliform ER virus (PBERV), are not thought to be associated with MWP as there is a lack of correlation between its presence and disease symptoms [10,11,12]. Members of two *Sadwavirus* species (family *Secoviridae*), pineapple secovirus-A (PSV-A) and PSV-B, were characterized from a pineapple germplasm accession and symptomatic commercial pineapples, respectively [3,13]. Although a clear association between symptoms and the presence of a badnavirus or secovirus in pineapple has not been shown, it is possible that a synergistic effect with the unrelated PMWaVs may induce severe symptoms of MWP.

An important resource of genetic material from several *Ananas* species, known as the HANA collection, is maintained at the United States Department of Agriculture, Agricultural Research Service (USDA-ARS), Tropical Plant Genetic Resources and Disease Research (TPGRDR) at the Daniel K. Inouye U.S. Pacific Basin Agricultural Research Center (PBARC) in Hilo, Hawaii, USA. The TPGRDR at PBARC maintains 186 active pineapple accessions as potted plants and in tissue culture https://www.ars.usda.gov/pacific-west-area/hilo-hi/daniel-k-inouye-us-pacific-basin-agricultural-research-center/tropical-plant-genetic-resources-and-disease-research/docs/main/ (accessed on 27 February 2020). Pineapple germplasm includes mostly *A. comosus* var. *comosus* accessions, but also includes other *A. comosus* cultivars like var. *ananassoides*, var. *erectifolius*, var. *bracteatus*, and some *Ananas* hybrids. Accessions of *Pitcairnia* sp., *Chevalvara stephanophora*, and *Bromelia factuosa* are also part of the HANA collection and have global geographic origins, including Africa, America, Asia, Europe, and the Pacific basin.

Viruses in pineapple plants may be present in low concentrations and as mixed infections and can exhibit non-specific symptoms [2,14]. Thus, reliable and specific detection tests are crucial to determine the virus status of pineapple propagules and control pineapple decline due to the cumulative effects of virus infection. Diagnostic methods for pineapple viruses currently available involve serological and molecular methods, including PCR and RT-PCR for the latter. Commonly, PCR-based tests are sensitive yet restricted to detecting a limited number of molecular variants. A genomic diversity of ampeloviruses, badnaviruses, and sadwaviruses infecting *Ananas* spp. has been proposed before previously [11,13,14,15,16,17]).

The use of high-throughput sequencing (HTS) has also been applied to explore the virome of vegetatively propagated crop germplasm, including berry crops [18], strawberry [19], grapevine [20,21], and pistachio [22]. These HTS studies have been useful in clarifying disease etiology, characterizing new viruses, and associating virus presence with symptom development.

In this study, HTS was used to further characterize the virome of 24 accession plants from the germplasm collection of *Ananas* spp at the USDA-ARS TPGRDR at PBARC. Bioinformatic analyses that included analysis of data mined from previous HTS studies on pineapple in China furthered the study of genetic diversity of viruses known to infect pineapple. We have also identified two new sadwaviruses, plus new molecular variants of sadwaviruses (PSVs) and PMWaVs. The complete genome sequence of the badnavirus PBERV was determined. Recombination events and virus diversity analyses allowed the development of PCR-based detection methods for indexing virus members of several species within the genera *Ampelovirus*, *Sadwavirus*, and *Badnavirus* that infect pineapple germplasm collection housed at USDA ARS.

## 2. Materials and Methods

### 2.1. Plant Material and RNA Extraction

A total of 65 *A. comosus* accessions, corresponding to the cultivars *comosus*, *erectifolius*, and *ananassoides*, and an *Ananas* hybrid maintained at the USDA, ARS, DKI-US-PBARC, TPGRDR (Hilo, HI, USA) were selected for analyses according to their geographic origin (Figure S1). Leaf samples from twenty-four of the 65 accessions were originally submitted to RNA-sequencing (RNA-seq) to clarify the pineapple virome. Virus indexing was completed on the 65 accessions (Table S1). Total RNA was extracted from the basal portions of the leaves from each pineapple accession sample using the Spectrum^TM^ Plant Total RNA Kit (Sigma-Aldrich, MO, USA), according to the manufacturer’s instructions. Quantity and quality of RNA were measured using NanoDrop^TM^ 2000 Spectrophotometer (Thermo Fisher Scientific, Waltham, MA, USA).

### 2.2. RNA-Sequencing and Viral Genome Assembly

RNA samples extracted from the 24 accessions were mixed in equimolar ratios into three pools of RNA termed composite sample 1 (CS1), CS2, and CS3. Each CS contained eleven, nine, and four pineapple accessions, respectively (Figure S1). Pooled RNA samples were sent to the Genomics and Bioinformatics Shared Resource (GBSR) at the University of Hawaii Cancer Center for RNA-seq. Total RNA was subjected to ribosomal RNA depletion using RiboMinus^TM^ Plant Kit (Thermo Fisher Scientific, Waltham, MA, USA). Library preparation was performed with an Illumina library preparation (TruSeq RNA Library Prep Kit; Illumina, San Diego, CA, USA), and sequenced in an Illumina NextSeq 500 System that provided approximately 63 million reads of 75-bp paired-end for each library composite sample. Virome analysis was carried out as described by Green et al. [16] with slight modifications. Trimming the raw reads to improve quality was performed with Trimmomatic [23]. Quality reads were then mapped to a draft pineapple genome available at http://ftp.ensemblgenomes.org/pub/plants/release-53/fasta/ananas_comosus/dna (accessed on 31 May 2020). Non-host reads were assembled into contigs using Trinity v2.9.1 [24] and rnaSPAdes v3.15.3 [25]. Assembly of viral genomes was performed using two parallel approaches: (i) BLASTx searches were adopted on assembled contigs against reference viral databases; and (ii) generated contigs were mapped against virus references using the Geneious mapper implemented in Geneious version 2022.1.1 [26] to confirm read coverage and genome completion. Open reading frames (ORFs) were analyzed with ORF Finder https://www.ncbi.nlm.nih.gov/orffinder/ (accessed on 29 July 2021) and protein-conserved domains were identified using the NCBI Conserved Domain search tool https://www.ncbi.nlm.nih.gov/Structure/cdd/wrpsb.cgi (accessed on 29 July 2021). For newly discovered sadwaviruses, cleavage sites of the polyprotein (P) were analyzed by multiple protein alignments with other sadwavirus members within the *Cholivirus* subgenus. All tools described above provided the annotation for viral identification.

### 2.3. HTS Validation

To confirm that the identified viral contigs were not artifacts, RT-PCR assays were carried out in 20 μL reactions following the protocols developed previously for detection of PMWaV-1, -2, -3, -5, and -6 [3,8,9,14,16]; badnaviruses PBCOV and PBERV [11,15]; and the sadwaviruses PSV-A and -B [3,13] (Data not shown). Positive controls for RT-PCR assays included cDNA derived from commercial field samples previously identified as harboring target viruses by RT-PCR and Sanger sequencing.

### 2.4. Identification of a Novel Species within the Sadwavirus Genus from Public Domain Ananas spp. Transcriptome Datasets

Data mining to assess the presence of uncharacterized virus species in publicly available *Ananas* spp. transcriptomic data was performed as described by Bejerman and colleagues [27,28]. In brief, amino acid sequences of the RNA-dependent RNA polymerase (RdRp), and coat protein (CP) of PMWaVs, polyprotein 1 (encoded by RNA1) and polyprotein 2 (encoded by RNA2) of PSVs, and the CP and the reverse transcriptase (RT) of pineapple-infecting badnaviruses were used as query in tBlatsn searches against *Ananas* (taxid: 4614) in transcriptome shotgun assemble (TSA) databases. TSA accessions of virus homolog sequences that shared less than 75% identity were downloaded for further analysis. Bioinformatic analyses including de novo assembly, contig annotation, and genome extension, were performed as detailed above.

### 2.5. Study of Intra-Species Recombination and Genomic Diversity

To estimate the divergence and recombination of the retrieved viral sequences, multiple alignments of nearly complete genome sequences were performed using the MUSCLE algorithm [29] implemented in MEGA11 [30]. Sequences used for analysis were represented by nearly complete genome excluding the 5′ and 3′ ends. Viral sequences of the viral agents retrieved from the 24 accessions pooled in CS1, CS2, and CS3 were aligned with their corresponding relatives including ampeloviruses, sadwaviruses, and badnaviruses. The occurrence of potential recombination events specific to the members of each virus species was evaluated using seven methods: RDP, GENECONV, Chimaera, MaxChi, BootScan, SiScan, and 3Seq implemented in RDP4 [31]. Recombination events supported by robust statistics for three of the seven algorithms available in the RDP4 software were accepted as reliable predictions.

Intra- and inter-group diversity analyses were accomplished by phylogenetic analysis of the alignments of almost nearly complete genome sequences from the retrieved viral sequences. PBCOV and PBERV diversity analysis was not performed for the badnaviruses, due to insufficient number of sequences. For sadwaviruses, the diversity of RNA1 and RNA2 sequences was analyzed independently. The Maximum Likelihood (ML) method based on the General Time Reversible (GTR) model with 1000 bootstrap replicates was used with gamma distributions of +G and +G + I for ampeloviruses and sadwaviruses, respectively.

### 2.6. Phylogenetic Analyses to Explore Inter- and Intra-Species Relationships

Sequences of representative members from each group were selected for phylogenetic analysis using the ML method with 1000 bootstrap replicates. The ClustalW [32] algorithm was used in multiple sequence alignments of complete protein sequence homologs of the heat shock protein 70 (HSP70) for ampeloviruses, the RT-RH1 region between the RT and ribonuclease-H (RNase H) for badnaviruses, and the Pro-Pol region between the protease (Pro) and the RdRp for sadwaviruses. Multiple sequence alignments were generated in MEGA11 [30]. The best models of protein evolution for the HSP70 (GTR+G), RT-RH1 (GTR+G+I), and Pro-Pol (Le Gascuel [LG)+G+I]) were used.

### 2.7. Evaluating and Optimizing RT-PCR Assays for Sensitive Detection of Viruses Infecting Pineapple

Previously developed primer sets for PCR-based assays targeting viruses infecting pineapple [3,8,11,14,15,16] were evaluated in silico to determine their capacity to detect all species variants reported in this study based on the thermodynamic parameters described by Arif and Ochoa-Corona [33]. Additionally, this study evaluated potential primer mismatches in the newly characterized virus sequences. Nucleotide sequences of the HSP70 (ampeloviruses), RNaseH (badnaviruses), RdRp (sadwaviruses, RNA-1), and CP (sadwaviruses, RNA-2) were aligned to identify conserved regions that could be used for specific and broad detection of all virus sequence variants. Primaclade software [34] was used to identify universal virus species-specific primer sets targeting conserved regions among the multiple viral sequence variants identified in each species group. Previous and newly developed sets of primers were subjected to BLAST analysis to corroborate specificity using the primer-blast tool https://www.ncbi.nlm.nih.gov/tools/primer-blast/ (accessed on 12 August 2021). Validation of newly developed RT-PCR assays was performed by comparing results obtained using the detection methods previously reported [3,8,14]. RT-PCR assays were carried out using GoTaq^®^ DNA polymerase (Promega, WI, USA) in reactions of 20 µL and 0.5 µM as the final primer concentration. cDNA was synthetized using 0.1 μg/μL of total RNA, and 2 uL were added as template in RT-PCR reactions. The optimum annealing temperature was determined using a gradient PCR assay with annealing temperatures from 48 to 60 °C using cDNA from at least two pineapple accessions as templates. PCR amplicons were gel-extracted, purified, and bi-directionally sequenced.

All RT-PCR conditions were as follows, with modifications in the annealing temperature according to the thermodynamics of each primer set (Table 1) and results from gradient PCR: initial denaturation at 94 °C for 5 min, 35 cycles at 94 °C for 40 s, X °C for 30 s, and 72 °C for 45 s, with a final extension at 72 °C for 10 min. “X” represents the annealing temperature used for each primer set which varied from 48 °C to 55 °C (Table 1).

## 3. Results

### 3.1. Characterization of the Virome in Pineapple Germplasm Allows the Discovery of Two Novel Sadwavirus Species

The 24 pineapple accessions from different geographic origins representing countries from the Americas, Africa, Asia and Europe were subjected to RNA-seq. RNAs for individual accessions were randomly pooled into three composite samples (CS): Brazil, Colombia, Guatemala, Mexico, Panama, Paraguay, Puerto Rico, Trinidad, and Venezuela (CS1); Barbados, India, Indonesia, Jamaica, Philippines, USA [Hawaii (HI) and Florida (FL)], Taiwan, and Vietnam (CS2); and the Democratic Republic (DR) of Congo, Samoa, South Africa, and Portugal (CS3) (Figure S1). Illumina-sequenced paired-end 75-bp read length was 63.9 M for CS1, 63.2 million for CS2, and 67.7 million for CS3.

Bioinformatic analyses revealed four previously characterized members of the PMWaV complex (*Ampelovirus* genus, *Closteroviridae* family), three sadwaviruses (two previously reported and one novel species), and two badnaviruses, for a total of nine viruses detected in pineapple germplasms. The identified viral sequences within each species are described in Table S1.

#### 3.1.1. Ampeloviruses

At least one sequence representing the nearly complete genomes of PMWaV-1, PMWaV-2, PMWaV-3, and PMWaV-6 were recovered from each of the CS data sets when contigs and raw data were aligned to the reference genomes available in the GenBank database [4,6,8]. PMWaV-5 sequences were not recovered from any of the CS datasets after analysis and mapping against partial sequences characterized from Australian pineapple [14]. For CS1, a total of 28,082 reads were mapped against PMWaV-2, representing 70.1% of the total reads for ampeloviruses (39,602 reads). The same scenario was observed for CS2 and CS3 where reads mapped for PMWaV-2 represented 60.3% (37,131 out of 61,845), and 69.2% (45,136 out of 65,215), respectively (Table S1). These ratios suggest that PMWaV-2 may be present at higher titers than other members of the PMWaV complex.

**Figure 1:**
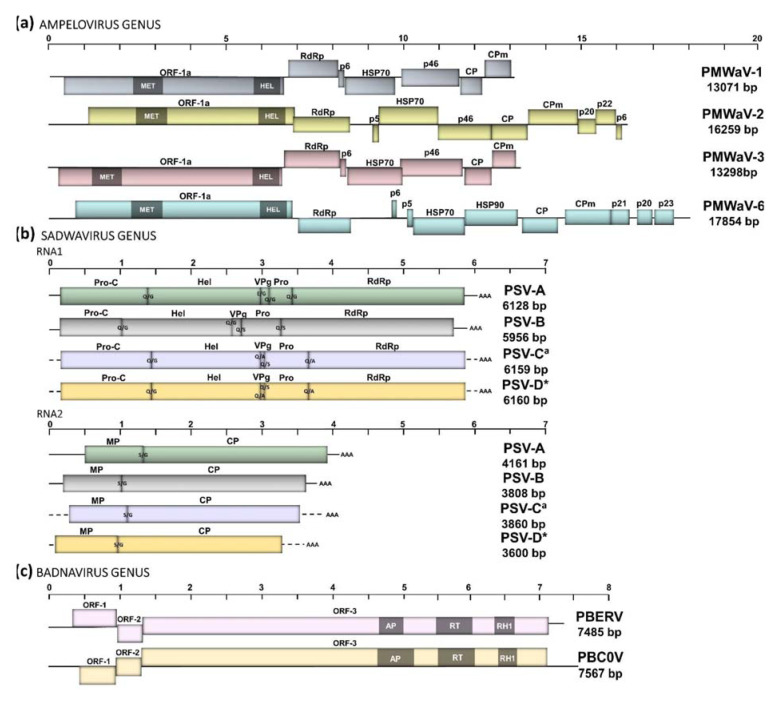
Genome organization of viruses infecting pineapple: (**a**) Genome organization of the pineapple mealybug wilt-associated virus (PMWaV) complex. Three classified and one yet to be classified *Ampelovirus* species have been characterized infecting pineapple: PMWaV-1, PMWaV-2, PMWav-3, and PMWaV-6, respectively. PMWaV-4 previously reported as another species was recently classified as a strain of PMWaV-1; (**b**). Genome organization of virus members infecting pineapple and classified within *Sadwavirus* species: pineapple secovirus A (PSV-A), PSV-B, PSV-C (^a^, new species infecting germplasm), and PSV-D (*, recovery from data mining). Vertical lines within the boxes represent putative cleavage sites; (**c**). Genome organization of virus members infecting pineapple and classified within *Badnavirus* species: pineapple bacilliform CO virus (PBCOV), and the report of the complete genome of pineapple bacilliform ER virus (PBERV). Abbreviations: MTR and HEL, methyltransferase and helicase domains of ORF-1a (replicase polyprotein); RdRp, RNA-dependent RNA polymerase; HSP70–90, heat shock protein 70 and 90; CP, coat protein; CPm, minor CP; p5, p6, p20, p21, p23, and p46 represent protein with their respective weights (kDa); Pro-C, protease factor; Hel, helicase; VPg, viral-linked protein; Pro, protease; MP, movement protein. Abbreviations of conserved protein domains: AP, retropepsin (pepsin-like aspartic protease); RT, reverse transcriptase; RH1, Ribonuclease H. Dashes lines in the genomes mean incomplete sequences at the 5′ and 3′ untranslated regions (UTR). Boxes represent identified open reading frames (ORF).

A multiple alignment using the amino acid sequences of HSP70 homologs to the PMWaV complex was used to study their genetic identities with member species within the genus *Ampelovirus* (family *Closteroviridae*). Overall, PMWaV-1 (13,071 bp) and PMWaV-3 (13,259 bp) were clustered with members of subgroup II, while PMWaV-2 (16,259 bp) and PMWaV-6 (17,854) both with longer genomes, clustered within subgroup I (Figure 1 and Figure S2). Although genomic organization at the 5′-genomic region encoding non-structural proteins (ORF1a and ORF2) was similar among all PMWaVs, additional proteins located at the 3′-genomic region in PMWaV-2 and PMWaV-6 were unique features of these larger ampeloviruses. Some proteins located in the 3′-genomic region of PMWaV-1 and -2 were tested for possible roles as gene silencing suppressors of the plant defense mechanism in transient assays [35].

#### 3.1.2. Sadwaviruses: Two Novel Species

In addition to ampeloviruses, the genome sequences of three members of the *Sadwavirus* genus (*Secoviridae* family) were retrieved from the three CSs datasets. Variants of PSV-A were found in CS1 and CS2 only, while variants of PSV-B were recovered from all three datasets (CS1, CS2, and CS3; Table S1). In addition to the previously reported PSV-A [3] and PSV-B [13], two RNA molecules representing a putative new *Sadwavirus* species were retrieved from CS2. Polyprotein 1 encoded by RNA1 (4474 bp) was predicted to be cleaved by proteases at four sites, while a polyprotein 2 encoded by RNA-2 (3860 bp) was predicted to be cleaved at only one site (Figure 1). This putative new virus shared 34.8 and 40.8% protein identity with the Pro-pol homolog regions of PSV-A and PSV-B, respectively. The name pineapple secovirus C (PSV-C) is proposed for this new sadwavirus infecting *Ananas* germplasm.

Furthermore, two contigs were retrieved by data mining from a TSA dataset associated with the bio-project PRJNA356904, corresponding to the transcriptome data from an *Ananas comosus* sample collected from China (unpublished) and deposited in the NCBI database. RNA1 from this putative virus was predicted to code for an RNA that is 6160 bp in length, while RNA2 codes for a 3600-bp in length (Figure 1). Similar to PSV-C, polyproteins 1 and 2 encoded by RNA1 and 2 were predicted to be cleaved at four sites and one site, respectively. This putative novel virus shared protein identities of 35, 41.3, and 73.2% to their homologs from the Pro-pol region of PSV-A, PSV-B, and PSV-C, respectively. The name pineapple secovirus D (PSV-D) is proposed for this putative sadwavirus which genome retrieved from a public TSA dataset.

A multiple alignment of the amino acid sequences of the polyproteins coded by RNA-1 and-2, with homologous members in the *Sadwavirus* genus, was done to study their evolutionary relationships. The putative new viruses, PSV-C and PSV-D, grouped with their relatives in the *Cholivirus* subgenus cluster (Figure S2). Dipeptides were predicted to cleave polyprotein 1 of the novel sadwaviruses PSV-C and PSV-D into five putative proteins: protease factor (Pro-C), helicase (Hel), viral-genome linked protein (VPg), protease (Pro), and RdRp (Figure 1).

Polyprotein 2 of the two novel sadwaviruses was predicted to be cleaved by one dipeptide into two putative proteins: coat protein (CP) and movement protein (MP). The most probable cleavage sites in RNA-1 and RNA-2 for sadwaviruses infecting *Ananas* spp. are shown in Figure 1. Amino acid sequences within the Pro-Pol conserved region of the virus members infecting pineapple and classified within two novel *Sadwavirus* species were used to compare their relatedness with other members of the *Secoviridae* family. PSV-C and PSV-D clustered together with other members classified within the subgenus *Cholivirus* (genus *Sadwavirus*, family *Secoviridae*), including the previously characterized PSV-A and PSV-B (Figure S2). Protein identities of the conserved Pro-pol region of below 80%, and phylogenetic analyses, suggest that PSV-C and PSV-D are members of two novel species belonging to the *Sadwavirus* genus. Sequence comparisons based on the coding region of P1 and P2 also support that PSV-C and -D are novel viruses infecting *Ananas* (Tables S2 and S3).

#### 3.1.3. Badnaviruses: Report of the Complete Genome of PBERV

After following bioinformatic analyses, the complete genome sequences of two badnaviruses (family Caulimoviridae) were retrieved from the CSs datasets. Genome sequences of the previously reported PBCOV [11,15] were retrieved from datasets CS1 and CS2. The complete genome of the PBERV was retrieved from dataset CS3. Previously, only a partial sequence of PBERV had been reported by Gambley and collaborators [11]. Starting at the putative plant initiator tRNA^MET^-binding site (TGGTATCAGAGC) three putative ORFs were identified for PBCOV and PBERV and named P1, P2, and P3. This genomic organization is typical of members within the *Badnavirus* genus [36]. P3 is a polyprotein coding for structural and non-structural proteins. Analysis of conserved domains in the P3 polyprotein revealed the presence of the retro-pepsin, pepsin-like aspartic protease (AP; cl11493), reverse transcriptase (RT; pfam00078), and the RNase H1 (RH1; cl39037) in both PBCOV and PBERV (Figure 1). In addition, a conserved domain with recombination and DNA strand exchange inhibitor (cl29770, PRK00409 superfamily) function was detected in PBCOV from CS1. The domain was located between nucleotide positions 4492 and 4749 in the P3 ORF. No conserved domains were found for the viral movement protein (VMP) in PBCOV or PBERV.

Multiple nucleotide sequence alignments of the RT-RH1 region between the homologues of RT and RNase H from PBCOV and PBERV were performed. Phylogenetic analyses placed PBCOV and PBERV in a cluster with other members of the genus *Badnavirus* (Figure S2).

### 3.2. Inter- and Intra-Species Diversity and Recombination in Members of the PMWaV Complex

To understand the evolutionary tendencies of the virus members infecting *Ananas* spp. and that are classified within the genus *Ampelovirus*, we evaluated (i) inter-species diversity based on genome nucleotide identity and (ii) recombination events at the intra-species level. For in silico analysis, a reference virus isolate was included from the available data published by Green and collaborators [16]. In addition, an isolate retrieved from RNA-seq data of samples collected in 2019 from commercial fields in Hawaii was included in the analysis and referred to as MWP [9].

BLASTn annotation of the PMWaVs sequences revealed genetic diversity within members of the PMWaV complex. PMWaV members within subgroup II (PMWaV-1 and -3) were predicted to be more diverse at the nucleotide level than members within subgroup I (PMWaV-2 and -6). For PMWaV-1 and PMWaV-3, five genome sequences of each virus were retrieved by in silico analysis from the three CS. At least one sequence was recovered from each CS. Sequences of defective RNAs of PMWaV-1 and PMWaV-3 were also found in the CS2 dataset, but sequences of new strains of the same viruses were found in the CS3 dataset (Table S1). Nucleotide identities ranging from 84.3% to 98.1% were observed for PMWaV-1 sequences when compared with the reference isolate MN539276.

**Figure 2:**
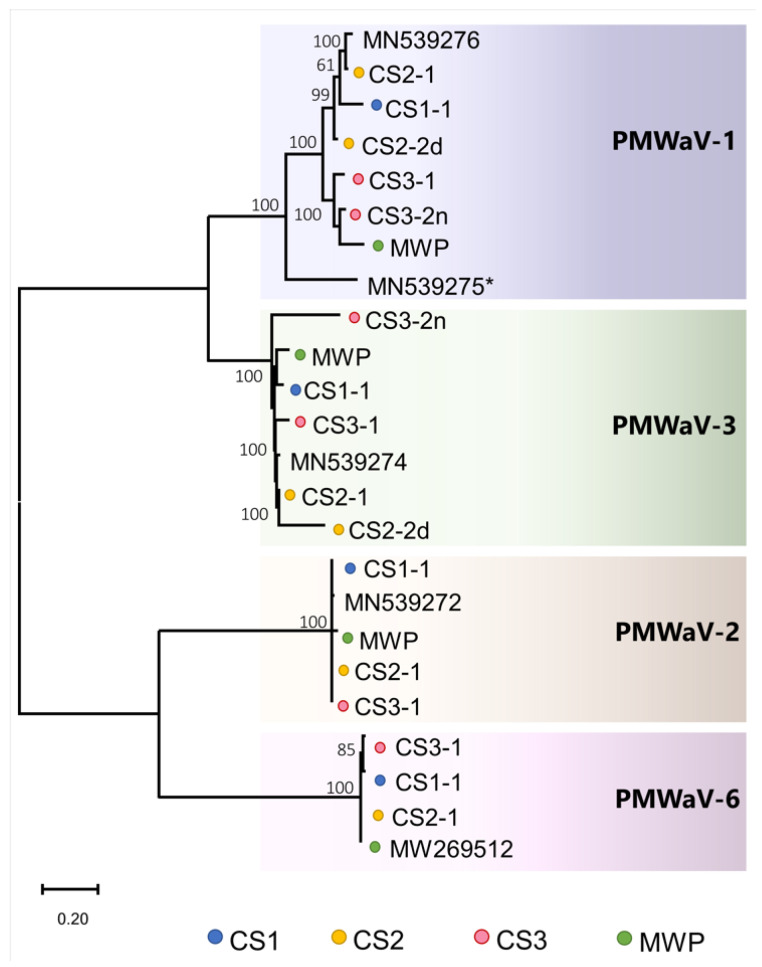
Phylogeny and molecular diversity of pineapple mealybug wilt-associated viruses (PMWaVs) infecting *A. comosus* based on nearly complete sequence comparison: PMWaV-1 and -3 sequences were more divergent when compared to PMWaV-2 and -6 sequences. New sequence variants were retrieved from germplasm of *Ananas* spp. for both PMWaV-1 and -3 (CS3-2n). MN539275 * represents strain 4 of PMWaV-1, which was previously known as PMWaV-4. MWP represents sequences retrieved from samples collected from mealybug wilt of pineapple (MWP)-symptomatic plants in commercial pineapple fields in 2019 [9].

Three main clusters were observed in the PMWaV-1 phylogeny. One clade contained CS1 and CS2 PMWaV-1 variant and the reference PMWaV-1 isolate. Interestingly, the new PMWaV-1 strain (CS3-2n) clustered with a PMWaV-1 variant retrieved from a separate RNA-seq dataset from MWP-symptomatic field samples collected in 2019 in Hawaii [9]. Both isolates grouped in a cluster containing a PMWaV-1 variant from CS3. Strain 4 of PMWaV-1, previously known as PMWaV-4, clustered in a divergent monotypic clade (Figure 2 and Figure S2).

**Figure 3:**
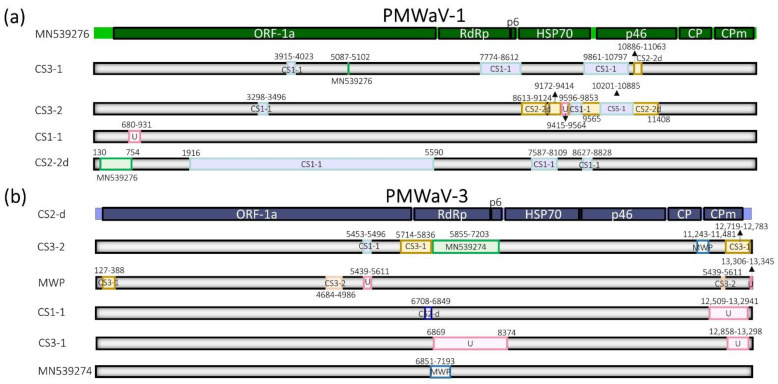
Map of recombination patterns and parental lineages of pineapple mealybug wilt-associated viruses-1 (PMWaV-1; (**a**,**b**) PMWaV-3: The major parents are represented in light shade, and the minor parents are represented in colored boxes. Events highlighted in light gray in the table were not considered for the analysis as they did not provide the support of at least three out of the seven algorithms available in the RDP4 software. Abbreviations: ORF-1a, replicase polyprotein; RdRp, RNA-dependent RNA polymerase; HSP70, heat shock protein 70; CP, coat protein; CPm, minor CP; p6 and p46 represent protein with their respective weights (kDa); U, unknown.

At least 16 independent recombination events were predicted among the PMWaV-1 variants. Most of the events were found across the quintuple gene block, and a few were found in the ORF1a region. No recombination events were found for the reference PMWaV-1 sequence MN539276. CS3-1 and CS3-2 sequences retrieved from CS3 were found as possible major parental sequences for all the recombination events of the four putative recombinants found (Figure 3a).

Nucleotide identities ranging from 74.0 to 96.9% were found for PMWaV-3 sequences compared to the reference isolate MN539274. Three main clusters were formed in the PMWaV-3 phylogeny. The new variant sequence retrieved from CS3 (CS3-2n) clustered in a monotypic lineage, whereas the CS1 variant grouped with the PMWaV-3 isolate retrieved from a dataset generated from MWP-symptomatic plants collected in 2019 from Hawaii [9]. The third cluster contained PMWaV-3 sequences retrieved from the three CS as well as the reference strain MN539274 (Figure 2).

Fourteen recombination events were predicted among the PMWaV-3 variants. These events were mostly identified across two regions: one region contained ORF1a, RdRp, and the p6; and the other contained the CP and minor coat protein (CPm). No recombination events were found for the defective RNA of PMWaV-3 from CS2. Several PMWaV-3 variants were identified as potential major and minor parental sequences for the putative recombinants identified (Figure 3b).

For ampeloviruses belonging to subgroup I (PMWaV-2 and PMWaV-6), genome comparison with their reference sequences (MN529272 and MW269512) revealed almost no differences at the nucleotide level. Identities from 98.7 to 99.7% were observed for PMWaV-2 variants, and 95.1 to 98.4% for PMWaV-6 variants. No distinctive clusters were found in the phylogeny of both viruses (Figure 2). No recombination events were found among PMWaV-2 variants, while a single recombination event was predicted in the PMWaV-6 variant retrieved from CS1 (CS1-1). This recombination event was further identified as a deletion in a non-coding region between the ORFs corresponding to the RdRp and p6 proteins. The potential major parental sequence for CS1-1 was the reference isolate of PMWaV-6 MW269512 (Figure S3).

### 3.3. Recombination Drives Diversity and Evolution of Sadwaviruses Infecting Ananas spp.

To study the diversity of PSVs, phylogenetic and recombination analyses were conducted on the recovered sequences of these viruses that represented most of the complete genome. PSV-A and PSV-B sequences previously reported by Larrea and collaborators [3] (Larrea et al., accepted for publishing) were used as references. PSV-A and PSV-B sequences recovered from RNA-seq data from MWP-symptomatic fields [3] were also included and referred to as MWP. The BLASTn annotation of these sequences revealed the presence of genetic diversity in the PSVs classified within the four species of the *Sadwavirus* genus (*Cholivirus* subgenus). Analyses of RNA1 and RNA2 from sadwaviruses were done separately since the number of retrieved sequences for these two RNA molecules differed for PSV-B.

**Figure 4:**
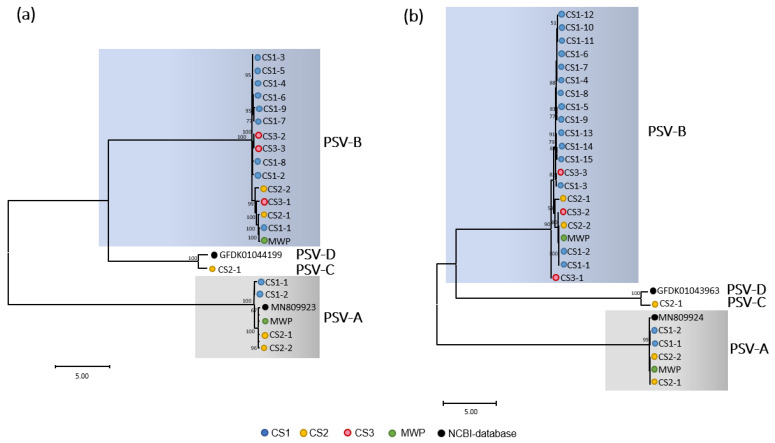
Phylogeny and molecular diversity of sadwaviruses infecting *A. comosus* based on nearly complete sequence comparison: The Phylogeny was inferred based on almost complete genome sequences of (**a**) RNA1 and (**b**) RNA2. pineapple secovirus (PSV)-A, -B and -C sequences were retrieved from *Ananas* germplasm and field samples collected in Hawaii. The sequences of PSV-C were retrieved in a dataset from China mined from the transcriptome shotgun assembly (TSA) database.

Genetic diversity was observed in the RNAs of both PSV-A and PSV-B. Since only one sequence each was retrieved for PSV-C and PSV-D, intra-species diversity was not analyzed. Analysis of the phylogenetic relationships among sequences representing nearly complete genomes clustered the sequence variants of members of each *Cholivirus* species into two very distinctive clades for PSV-A and -B. PSV-A sequence variants formed a monotypic lineage that branched from a clade containing PSV-B, -C, and -D sequences (Figure 4). These phylogenetic relationships may suggest that PSV-A is the most closely related virus to a cholivirus ancestor infecting *Ananas*. Genetic diversity in RNA1 and 2 of PSV-A is reflected in nucleotide identities ranging from 78.7 to 91.0% and from 86.3 to 89.2%, respectively, compared to the reference sequences (MN809923 and MN809924).

**Figure 5:**
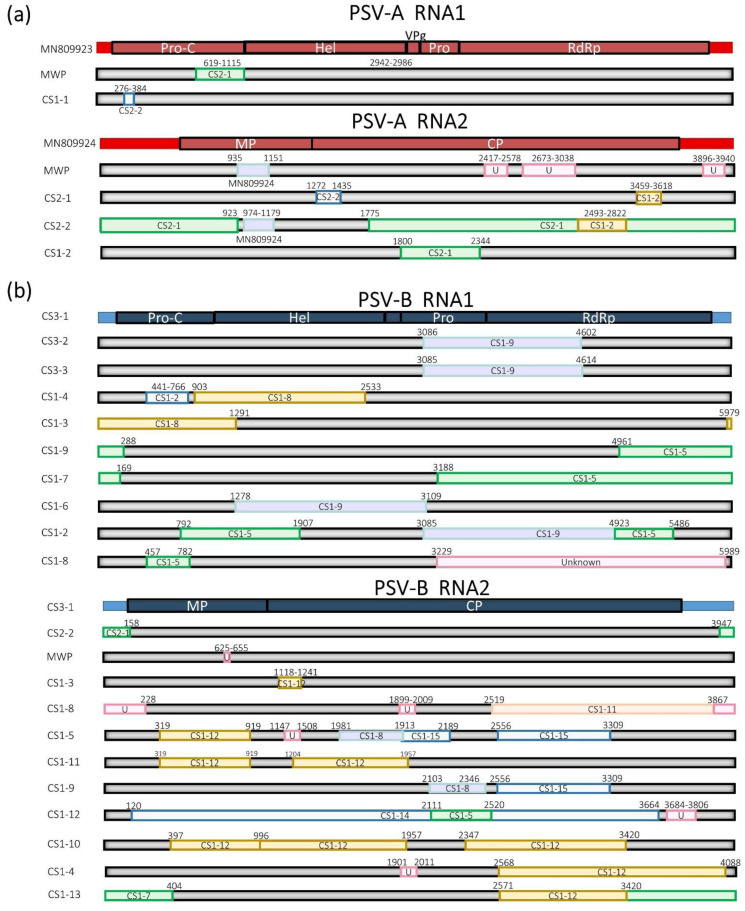
Map of recombination patterns and parental lineages of pineapple secovirus (**a**); PSV-A and (**b**) PSV-B: Potential recombination events on RNA-1. B. Potential recombination events on RNA-2. The major parents are represented in the light shade, and the minor parent in colored boxes. Potential recombination events from RDP software. Events highlighted in light gray in the table were not considered for the analysis since they did not provide support for at least three out of the seven algorithms available in the RDP4 software. Abbreviations: Pro-C, protease factor; Hel, helicase; VPg, viral-linked protein; Pro, protease; RdRp, RNA-dependent RNA polymerase; MP, movement protein; CP, coat protein; U, unknown.

PSV-A sequences grouped in clusters containing sequences originating from the same CS for both RNAs. However, the sequence variants for both RNAs of PSV-B grouped into several clusters that contained a mixture of sequences that originated from the three CSs (Figure 4). For PSV-A, four sequences, two each for RNA1 and RNA2 were retrieved from CS1. The same number of sequences were retrieved from CS2, but no PSV-A sequences were recovered from CS3. Two and five recombination events were predicted for RNA1 and 2, respectively. Both recombination events in RNA1 were predicted in the Pro-C coding region (Figure 5a), whereas the five recombination events were distributed along the RNA2 genomic sequence (Figure 5a). No recombination events were found for RNA1 or 2 of the reference isolate of PSV-A (MN809923 and MN809924).

Fourteen sequences representing the nearly entire genome sequences of PSV-B RNA1 were retrieved: nine from CS1, two from CS2, and three from CS3 (Table S1). Twenty sequences representing PSV-B RNA2 molecules were also recovered: 15 from CS1, two from CS2, and three from CS3 (Table S1). Genetic diversity in the PSV-B sequence variants was represented by nucleotide identities between 76.3 to 94.4% for RNA1, and 70.6 to 93.4% for RNA2. Ten and 18 potential recombination events were identified for PSV-B RNA1 and RNA2, respectively. For both RNAs, the potential recombination events were located across their entire genomic sequences (Figure 5b). Most identified molecular sequence recombinants were found in CS1 samples that originated from countries in the Americas (Figure S1).

Phylogenic analysis placed the putative new choliviruses, PSV-C and -D, in the same clade, suggesting their close relationship (Figure 4). The PSV-C genome sequence was recovered from CS2, in which accessions came from countries mainly located in Asia but with some in the Americas (Figure S1 and Table S1). No recombination events were analyzed for PSV-C and -D since they represented unique samples and no other sequences of their type were available.

### 3.4. Improvement of RT-PCR Detection Methods and Virus Indexing of Ananas Germplasm

New RT-PCR detection methods were developed for members of the ampelovirus (PMWaV-1, -2, -3, and -6), badnavirus (PBCOV and PBERV), secovirus (PSV-A, -B, -C, and -D) genera reported in this study infecting pineapple germplasm. Oligonucleotides were designed based on diverged sequences in otherwise conserved sequence regions characteristic of each genus to detect and distinguish each virus species specifically. For variable regions, degenerate bases were included to avoid the need for multiple forward and reverse primers (Table 1).

PCR conditions were optimized using gradient PCR with a range of melting temperatures (°Tm) from 48 °C to 60 °C using the cDNA from at least two germplasm accessions (Figure S4) as templates. Sanger sequencing of the PCR products and pairwise nucleotide comparisons confirmed the virus sequence identity of the amplicons generated by all virus-specific primer sets. All amplified sequences presented >98% nucleotide identity to the virus reference sequence obtained through HTS (data not shown).

To confirm the presence of viruses identified by RNA-seq, a total of 65 accessions from the pineapple germplasm (HANA collection) were selected for virus indexing using the above-described RT-PCR assays. The original 24 accessions submitted for RNA-seq were included in the 65 accessions panel used for virus detection (Table 2). Two accessions, HANA-64 from Brazil and HANA-91 from Trinidad, were identified as virus-free plants based on RT-PCR analysis.

Ampeloviruses were found to infect accessions collected from around the world. PMWaV-1 and -2 were the most prevalent and were found in about 66.0% (43) of the accessions. PMWaV-3 was the third most prevalent virus in 29 accessions (44.6%). PMWaV-6 was only identified in 7.7% of the 65 accessions tested. These five accessions containing PMWaV-6 had origins in Asia, the Americas, and South Africa. PMWaV-5, previously reported by Gambley and collaborators [14], was not detected in any accessions. These results were consistent with the RNA-seq data (Table 2).

Among the pineapple viruses described in this study from the *Sadwavirus* genus, PSV-A was detected in 33 accessions with 50.8% prevalence, followed by PSV-B with 26.2% (17 accessions), and PSV-C with 3.1% (2 accessions). PSV-A and -B were detected in accessions collected worldwide, while PSV-C was limited to Asia. PSV-D, a genome retrieved from TSA data from pineapple in China, was not detected in any tested accessions. Both badnaviruses, PBCOV and PBERV, were found equally prevalent at 41.5% (27 accessions) but had different distributions. PBCOV was detected in accessions from Asia, the Americas, and South Africa whereas, PBERV was limited to the Americas and Asia (Table 2).

The newly developed RT-PCR assays proved robustness since a significant large number of accessions tested positive for PMWaV-1 and -2, and PSV-A and -B. Additional validation of the improved RT-PCR assays was done by using the 24 accessions originally submitted to RNA-seq. These results were compared using literature primers for PMWaVs and PSV-A and -B (Table 2) [3,8,13].

## 4. Discussion

In the present work, RNA-seq, bioinformatic analyses, and RT-PCR assays were used to study the prevalence and diversity of viruses classified within the *Ampelovirus*, *Sadwavirus*, and *Badnavirus* genera that infect *Ananas* spp. germplasm. This is the first report of the virome in *Ananas* germplasm. Nearly complete genome sequences representing molecular variants of the previously characterized PMWaV-1, -2, -3 and -6, and PSV-A and -B, and PBCOV were recovered from three CS datasets. Nearly complete genome sequences from two new species within the *Cholivirus* subgenus (*Sadwavirus* genus), putatively named PSV-C and PSV-D, were also retrieved from CS2 and a dataset (GFDK01044199) mined from the TSA database, respectively (Figure 4). The TSA dataset corresponded to a pineapple sample collected in China. Likewise, this is the first time where the complete genome of PBERV is reported in the present study (Figure 1). Phylogenetic and recombination analyses suggested recombination in PMWaV-1, -3 and -6 (Figure 2, Figure 3 and Figure S3) and in RNA1 and 2 of PSV-A and PSV-B (Figure 3, Figure 4 and Figure 5). Intra-species recombination events of ampeloviruses and secoviruses have been shown previously [37,38].

Because viral sequences were retrieved from pooled samples (CS1–CS3), it was not possible to determine if sequence variants of the same virus co-exist in the same plant. However, potential recombination events found in different PMWaVs and PSVs isolates suggest that the recombination ability of the viruses infecting *Ananas* may play a role in virus evolution and perhaps in mealybug wilt of pineapple (MWP). MWP disease is the most economically important viral disease affecting pineapple production worldwide, but the disease etiology is not yet fully understood. In Hawaii, MWP symptoms are associated with infection by PMWaV-2 and mealybug feeding. This is different than Australia, where PMWaV-2 is not found at higher prevalence, but instead PMWaV-1 and PMWaV-3 are correlated with MWP symptoms [7,11]. This discovery has led to the hypothesis that uncharacterized viruses may be playing a role in the disease. Using RNA-seq, discovery of the ampelovirus PMWaV-6 and the two sadwaviruses, PSV-A and PSV-B, provides additional insight into novel viruses that may be involved in the etiology of MWP.

Identified potential recombination events in PMWaV-1, -3, PSV-A, and -B in non-structural and structural protein-coding regions further suggest that these events are not detrimental to these viruses and may promote the emergence of stable variants. A similar scenario was observed for grapevine leaf roll-associated virus 4, one of the viruses linked with grapevine leaf roll disease [37]. The discovery of new strains from CS3 and defective RNAs (dRNA) from CS2 for PMWaV-1 and -3, suggest virus recombination of these two members belonging to the subgroup II of the *Ampelovirus* genus. dRNAs from members within the genera *Crinivirus, Closterovirus* and *Ampelovirus* (family *Closteroviridae*) have been described previously. Their presence can (i) result in less virus accumulation, and milder symptoms, or (ii) induce severe symptoms associated with the disease [5,39,40,41,42]. dRNAs of PMWaV-1 have been previously reported in pineapple [5]. Even though genetic sequence variants and recombination have been reported for other ampeloviruses in subgroup I [43], an overall low genetic diversity was observed for PMWaV-2 and -6. These results are consistent with our recombination analyses where only one putative recombination event was identified in a non-coding region of PMWaV-6 (Figure S3).

PMWaV-1, -2, and PSV-A are the most prevalent viruses (>50%) infecting *Ananas* germplasm. Virome studies and indexing revealed that although the 24 *A. comosus* accessions were found in the same germplasm collection, they harbor different viral combinations perhaps due to differences in their geographic origins (Figure S1 and Table 2). Even though a smaller number of sequence variants were retrieved for PSV-A than from PSV-B, a major number of HTS reads mapped to the PSV-A genome, except for the CS3 dataset where no reads mapped to the PSV-A genome (Table S1). Surveys of commercial pineapple fields in Hawaii (Larrea-Sarmiento et al., 2019; Larrea-Sarmiento et al., 2021) revealed that PMWaV-2 is the most prevalent virus in MWP-symptomatic plants in Hawaii. On the other hand, PMWaV-1 and -3 are prevalent in both MWP-symptomatic and non-symptomatic plants in Hawaii. Plants displaying severe MWP symptoms can also be infected with PMWaV-6 and PSV-A (Larrea-Sarmiento et al., 2021).

It was previously proposed that closteroviruses use a group of “buffering system” strategies to protect their large RNA genomes from the host defense RNA silencing mechanism. The mechanism of these strategies includes gene-silencing suppressors, double-stranded RNA (dsRNA), subgenomic RNAs, and dRNAs [42]. The PMWaV-1 and -3 dRNAs found using HTS from the CSs may explain why they are highly prevalent viruses in both MWP-symptomatic and asymptomatic plants.

RNA-seq and virus-specific RT-PCR assays did detect the badnaviruses PBCOV and PBERV. Considering their potential virus genome integration (endogenous form) into the nuclear genome of pineapple with its genomic diversity, RT-PCR assays containing a DNase digestion step before cDNA synthesis enabled the distinction between endogenous and episomal forms [11,15]. The genomic diversity of PBCOV and its transmission by the gray pineapple mealybug, *Dysmicoccus neobrevipes* has been reported previously [15].

The use of RNA-seq coupled with ribodepletion enabled the identification of divergent genetic sequence variants of PMWaVs and PSVs and the characterization of a novel sadwavirus, PSV-C. Report of the complete genome sequence of PBERV was also achieved using RNA-seq. Moreover, data mining allowed the discovery of another putative sadwavirus, PSV-D, by bioinformatic analysis of a TSA dataset from China. Interestingly, PSV-D was not found infecting any of the virus index *Ananas* accessions in the USDA ARS pineapple germplasm collection. The genetic diversity observed in viruses infecting *Ananas* underscores the need for robust, sensitive detection methods that allow specific identification of several sequence variants of each virus. The developed RT-PCR assays proved capable of detecting different intra-species molecular variants and were also specific enough to differentiate them from their closely related species.

Furthermore, the implementation and improvement of RT-PCR diagnostic assays reported in this study is imperative for virus indexing and monitoring in the vegetably propagated pineapple germplasm and propagules. Newly developed RT-PCR assays were shown to be able to reliably detect molecular variants within the PMWaVs and PSVs compared to previously reported diagnostic RT-PCR assays (Table 2) [3,8,14]. These newly developed RT-PCR assays could be a fundamental tool in virus surveillance, especially for virus indexing of MWP-symptomatic and asymptomatic plants in regions where the etiology of MWP is not clearly associated with specific viruses.

## Figures and Tables

**Table 1:** Oligonucleotides used in the RT-PCR assays for the detection of viruses infecting *Ananas* spp.

Virus Genus	Virus Specie	Target	Sequence 5′–3′	Product Size (bp)	Length (bp)	Tm	Annealing PCR-Conditions ^a^	Reference
**Ampelovirus**	PMWaV-1	HSP70	GGTTGTTCTATAGGACCAGT	314	20	58	51 °C	This study
			GTRCCYCCTCCGAAATCGTA		20	60		
	PMWaV-2		CGAACTAGACTCATACGTGC	571	20	50	55 °C	This study
			CGGCTCATTAGTCACCTCCT		20	55		
	PMWaV-3		ACDTTGTGTTTCTCCCCTGG	272	20	60	51 °C	This study
			GATCCATCRACGGGACCRAT		20	60		
	PMWaV-5		CCCGGACGTGAATGATGAAG	672	20	55	50 °C	This study
			GTATTAGCTGCGCCCGTTCT		20	55		
	PMWaV-6		GGAGTTGCGGGTTGTCTTCC	450	20	64	55 °C	Larrea-Sarmiento et al., 2021
			ATAGCCCTCACCGGTACTCC		20	64		
**Sadwavirus**	PSV-A RNA1	RdRp	GTGACATTTTTCACRAAYTGGGA	659	23	55	48 °C	This study
			CTCTGCTGRCAYTGAGCAA		19	56		
	PSV-A RNA2	CP	GAATCGCGYAATATGGTGCA	544	20	58		This study
			ATCCTCCTGAGTCAGTRGGCAA		22	59		
	PSV-B RNA1	RdRp	TAYAACTGGGAYATYATGGCDTC	317	23	56	51 °C	This study
			CGCATTATDGCATACCARCT		20	56		
	PSV-B RNA2	CP	AGVATGGGAGCKTTCCACAT	415	20	58		This study
			GTGCANGARCCAGCTATRCT		20	58		
	PSVC-RNA1	RdRp	CTGCTACCCAACCACCAGAA	480	20	62	55 °C	This study
			CCATCACGGTCAAAGCAAATCC		22	58		
	PSVC-RNA2	CP	GTCTGTTGTCTCTGCCCTCA	539	20	62		This study
			TAYAAAGAACAACTGCCAGCAAC		23	56		
	PSVD-RNA1	RdRp	GAGAGACGGGAACAGGAACC	360	20	64	55 °C	This study
			TGCCCATCGGTAAGTCTCAC		20	62		
	PSVD-RNA2	CP	GCTAAATCCCGTTGTGAAGCC	657	21	58	51 °C	This study
			GCAACAGAAAGCTTGGAGTGG		21	58		
**Badnavirus**	PBCOV	RNaseH	ACAAGGACCTTCAAACWACA	631	20	56	51 °C	This study
			TCTTCATGCAGCATCATBAC		20	56		
	PBERV	RNaseH	GGAGTGAAGCAGGTGGACTT	463	20	62	55 °C	This study
			TYTCTGCGTCGATTGTGCTT		20	58		

^a^ RT-PCR assays were carried out as follows: initial denaturation of 94 °C for 5 min, 35 cycles of 94 °C for 40 s, X °C for 30 s, and 72 °C for 45 s, final amplification was 72 °C for 10 min. X represents the annealing temperature used for each primer set. PMWaV, pineapple mealybug wilt-associated virus; PSV, pineapple secovirus; PBCOV, pineapple bacilliform CO virus; PBERV, pineapple bacilliform ER virus; HSP70, heat shock protein 70; RdRp, RNA-dependent RNA-polymerase; CP, coat protein.

**Table 2:** Virus indexing of pineapple mealybug wilt-associated virus (PMWaV)-1, -2, -3 and -6, pineapple secovirus (PSV)-A, -B, -C and -D, pineapple bacilliform CO virus (PBCOV), and pineapple bacilliform ER virus (PBERV) on 65 *Ananas* spp. germplasm accessions maintained at the USDA-ARS TPGRDR at PBARC in Hilo, Hawaii.

				PMWaV				PSV						
Sample No.	HANA Accession	Origin	ID	-1	-2	-3	-6	-A	-B	-C	-D	PBCOV	PBERV	Summary
1	6	Taiwan ^2^	Tainung #9	+	+	+	−	+	+	−	−	−	−	1, 2, 3, A, B
2	12	DR. Congo	Congo	+	+	+	−	+	+	−	−	+	−	1, 2, 3, A, B, Co
3	13	American Samoa	Spanish Samoa	+	+	+	−	+	+	−	−	+	−	1, 2, 3, A, B, Co
4	14	Singapur	Pernambuco	+	+	+	−	+	+	−	−	−	+	1, 2, 3, A, B, Er
5	16	Barbados ^2^	Bermuda	−	+	+	−	+	−	−	−	+	+	2, 3, A, Co, Er
6	17	South Africa ^3^	Natal	+	+	+	−	−	+	−	−	+	−	1, 2, 3, A, B, Co
7	18	Taiwan	Mauritius	+	−	−	−	+	+	−	−	+	−	1, A, B, Co
8	20	Brazil	F 101	+	+	+	−	−	−	−	−	−	+	1, 2, 3, Er
9	21	Brazil ^1^	Abacaxi	+	+	+	−	−	−	−	−	−	−	1, 2, 3
10	22	Colombia	Santa Marta No. 1	+	+	+	−	+	−	−	−	+	+	1, 2, 3, A, Co, Er
11	23	Jamaica ^2^	Sam Clarke	+	+	−	−	+	−	−	−	+	+	1, 2, A, Co, Er
12	27	USA: HI, Oahu	Wild Kailua	+	+	+	−	−	+	−	−	−	−	1, 2, 3, B
13	31	Philippines ^2^	Black Antigua	+	+	−	−	−	−	−	−	−	+	1, 2, Er
14	33	Indonesia ^2^	Kendal	+	−	−	−	−	+	+	−	−	−	1, B, C
15	34	Guatemala ^1^	Monte Lirio	−	+	−	−	+	−	−	−	−	+	2, A, Er
16	35	India ^2^	Amalsad	+	−	−	−	−	−	+	−	−	−	1, C
17	36	Panama	Cowboy	−	−	−	−	+	+	−	−	−	+	A, B, Er
18	37	Mexico ^1^	Criolla	+	+	−	−	+	−	−	−	−	−	1, 2, A
19	38	Brazil	Wild Brazil	−	−	+	−	−	−	−	−	−	−	3
20	39	Philippines	Philippine Hybrid	−	+	−	−	+	−	−	−	−	−	2, A
21	41	Vietnam	Pho Lang Tuang	+	+	−	−	−	−	−	−	−	−	1, 2
22	46	Zaire ^3^	Sugarloaf	+	+	+	+	−	−	−	−	−	−	1, 2, 3, 6
23	48	Mexico	Mexican Criolla	+	+	−	−	−	−	−	−	+	−	1, 2, Co
24	49	Colombia ^1^	Pina Criolla	+	+	+	−	−	+	−	−	−	+	1, 2, 3, B, Er
25	52	Colombia	Papuri Vaupes Colombia	+	+	+	−	−	−	−	−	−	−	1, 2, 3
26	53	Samoa ^3^	British Samoa P1	+	−	+	−	−	−	−	−	+	−	1, 3
27	54	Western Samoa	British Samoa P5	+	−	+	−	−	−	−	−	−	+	1, 3, Er
28	60	Guatemala	Spanish Guatemala	+	+	+	−	−	+	−	−	−	−	1, 2, 3, B
29	63	Brazil	CB 2	−	+	−	−	+	−	−	−	+	+	2, A, Co, Er
30	64	Brazil	CB 5	−	−	−	−	−	−	−	−	−	−	-
31	65	Brazil	CB 6	−	+	+	−	+	−	−	−	+	+	2, 3, A, Co, Er
32	67	Brazil	CB 10	+	+	−	−	−	−	−	−	−	−	1, 2
33	68	Brazil	CB 11	−	−	−	−	−	−	−	−	−	+	Er
34	69	Paraguay	CB 15	+	+	−	−	−	−	−	−	−	−	1, 2
35	70	Brazil	CB 17	−	+	+	−	−	−	−	−	−	−	2, 3
36	71	Paraguay ^1^	CB 18	−	−	−	−	−	−	−	−	−	+	Er
37	72	Paraguay	CB 19	−	−	−	−	+	−	−	−	−	−	A
38	73	Paraguay	CB 20	−	−	−	−	−	+	−	−	+	−	B, Co
39	74	Paraguay	CB 21	+	+	+	+	−	−	−	−	+	−	1, 2, 3, 6, Co
40	75	Argentina	CB 23	+	+	+	−	+	−	−	−	−	−	1, 2, 3, A
41	88	Brazil	CB 71	−	−	-	−	+	−	−	−	+	−	A, Co
42	90	Brazil ^1^	Prazeres	−	+	+	+	+	+	−	−	+	+	2, 3, 6, A, B, Co, Er
43	91	Trinidad ^1^	Trinidad	−	-	−	−	−	−	−	−	−	−	-
44	92	USA: HI ^1^	Cayenne 666	+	+	−	−	+	−	−	−	+	+	1, 2, A, Co, Er
45	101	USA: HI, Lanai	Cayenne M 4 W	+	+	−	−	+	−	−	−	+	−	1, 2, A, Co
46	110	USA ^2^	Cayenne M 109-5	+	+	+	−	+	−	−	−	+	+	1, 2, 3, A, Co, Er
47	115	USA: HI, Oahu	Cayenne M 226 Nubby	+	+	+	+	+	−	−	−	+	+	1, 2, 3, 6, A, Co, Er
48	117	USA: HI, Maui ^2^	Cayenne M 35	+	+	−	−	+	−	−	−	+	+	1, 2, A, Co, Er
49	123	Panama	Red Spanish	+	−	−	−	+	−	−	−	+	+	1, A, Co, Er
50	124	Panama ^1^	Taboga	+	−	−	−	+	+	−	−	−	+	1, A, B, Er
51	135	Venezuela	Morada	+	−	−	−	+	−	−	−	+	+	1, A, Co, Er
52	136	Venezuela ^1^	Criolla	+	+	+	−	+	−	−	−	−	−	1, 2, 3, A
53	138	Venezuela	Pina Lisa	−	−	−	−	−	+	−	−	+	+	B, Co, Er
54	139	Portugal ^3^	Cayenne Azores	+	+	+	−	−	+	−	−	−	−	1, 2, 3, B
55	140	Vietnam	Pakse	+	+	−	−	+	−	−	−	−	−	1, 2, A
56	142	Vietnam ^2^	Den	+	+	−	+	+	−	−	−	−	−	1, 2, 6, A
57	143	Colombia	Pina De Castilla	+	+	−	−	+	−	−	−	+	−	1, 2, A, Co
58	145	Puerto Rico ^1^	Cabezona	−	+	−	−	+	−	−	−	−	−	2, A
59	149	Brazil	CB 33	−	−	+	−	−	−	−	−	−	+	3
60	163	Thailand	N91-05	+	+	+	−	+	−	−	−	−	−	1, 2, 3, A
61	164	Thailand	N91-06	+	+	−	−	−	−	−	−	+	−	1, 2, Co
62	167	USA: FL	32419	−	−	−	−	−	−	−	−	+	+	Co, Er
63	173	Bolivia	Short fruit #2	−	+	+	−	+	−	−	−	+	+	2, 3, A, Co, Er
64	175	Bolivia	Long fruit #2	−	−	−	−	−	+	−	−	−	+	B, Er
65	219	USA: HI		+	+	+	−	−	−	−	−	−	−	1, 2, 3
			**Total positives**	**43**	**43**	**29**	**5**	**33**	**17**	**2**	**0**	**27**	**27**	
			**Prevalence (%)**	**66.1**	**66.1**	**44.6**	**7.7**	**50.8**	**26.2**	**3.1**	**0.0**	**41.5**	**41.5**	

^1^ Accessions grouped in CS1 for RNA-seq, ^2^ Accessions grouped in CS2 for RNA-seq, ^3^ Accessions grouped in CS3 for RNA-seq, + Samples that tested positive with the new detection methods but negative using primers previously reported in the literature.

## Data Availability

All the sequence data that was generated in this study has been uploaded to the GenBank database.

## Supplementary Materials

The following supporting information can be downloaded at: https://www.mdpi.com/article/10.3390/pathogens11121470/s1, Figure S1: Geographic origin of *Ananas comosus* accessions used for RNA-sequencing and virus indexing; Figure S2: Phylogenetic analysis of viruses infecting *Ananas* spp.; Figure S3: Map of recombination patterns and parental lineages of pineapple mealybug wilt-associated viruses-6 (PMWaV-6); Figure S4: RT-PCR detection methods for viruses infecting pineapple; Table S1: Number of high-throughput sequencing (HTS) reads and depth of coverage values for the viral contigs retrieved from pineapple germplasm accessions; Table S2. Polyprotein 1 (P1) sequence nucleotide (lower left) and amino acid (upper right) percent identity comparisons between partial sequences of molecular variants of four species belonging to pineapple secoviruses (PSVs), genus *Sadwavirus*; Table S3. Polyprotein 2 (P2) sequence nucleotide (lower left) and amino acid (upper right) percent identity comparisons between partial sequences of molecular variants of the four species belonging to pineapple secoviruses (PSVs), genus *Sadwavirus*.
