# Supplementary material for: Genetic Diversity of Viral Populations Associated with Ananas Germplasm and Improvement of Virus Diagnostic Protocols
Source: Pathogens. 2022 Dec 5;11(12):1470. doi: 10.3390/pathogens11121470 (PMC9787488; doi:10.3390/pathogens11121470)
Supplement: Supplementary file 1 [file pathogens-11-01470-s001.zip › pathogens-2023210-SI.pdf]

pathogens

MDPI

Article

Genetic diversity of viral populations associated with *Ananas* germplasm and improvement of virus diagnostic protocols

Adriana E. Larrea-Sarmiento[1], Alejandro Olmedo-Velarde[1], Xupeng Wang[1], Wayne Borth[1], Ryan Domingo[2], Tracie K Matsumoto[2], Jon Y Suzuki[2], Marisa M Wall[2], Michael J. Melzer[1], John Hu[1]*

[1] Department of Plant and Environmental Protection Sciences, University of Hawaii, Honolulu, HI 96822, U.S.A.; aelarrea@hawaii.edu; aolmedov@hawaii.edu; xupeng@hawaii.edu; borth@hawaii.edu; melzer@hawaii.edu; johnhu@hawaii.edu

[2] United States Department of Agriculture, Agricultural Research Service, Daniel K. Inouye U. S. Pacific Basin Agricultural Research Center, Hilo, HI 96720, U.S.A.; ryan.domingo@usda.gov; tracie.matsumoto@usda.gov; jon.suzuki@usda.gov; marisa.wall@usda.gov

* Correspondence: johnhu@hawaii.edu, Tel.: (808) 956-7281

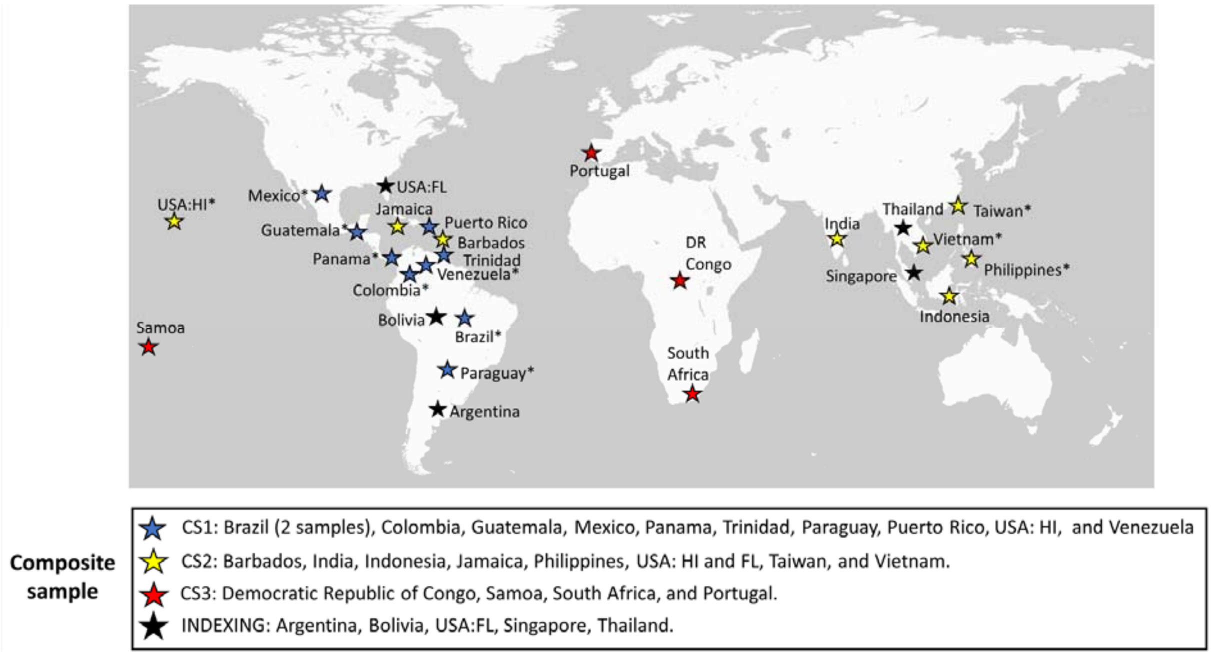

Figure S1: Geographic origin of *Ananas comosus* accessions used for RNA-sequencing and virus indexing: Country or region of origin of accessions submitted to RNA-sequencing: composite sample 1 (CS1), blue stars; CS2, yellow stars; CS3, red starts. Origin of accessions used only for virus indexing is represented by black stars. An asterisk (*) represents a geographic origin with more than one accession submitted to RNA-sequencing and virus indexing.

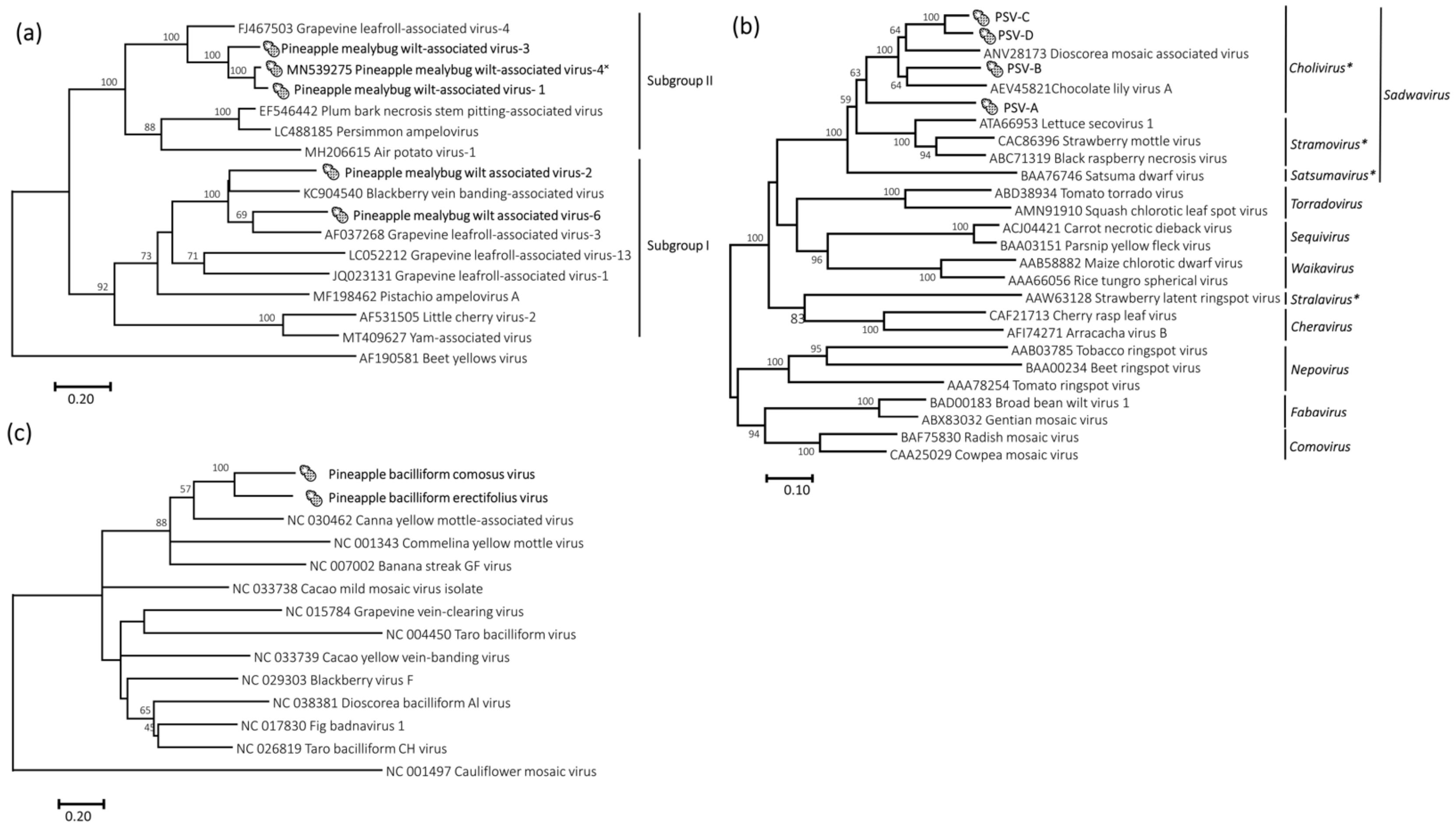

Figure S2. Phylogenetic analysis of viruses infecting *Ananas* spp.: (a) Phylogeny of the heat shock protein 70 (HSP70) of the pineapple mealybug wilt-associated virus (PMWaV) complex with homologs of members in the Ampelovirus genus. Four recognized *Ampelovirus* species have been characterized infecting pineapple: PMWaV-1, PMWaV-2, PMWav-3, and PMWaV-6. PMWaV-4, previously reported as another species, is currently known as variant of PMWaV-1. Beet yellows virus was used as an outgroup: **(b)** Unrooted phylogeny of the Pro-Pol protein region from members of the four *Sadwavirus* species characterized from pineapple with homologs of members within the family *Secoviridae*. Four sadwaviruses are reported

infecting pineapple: pineapple secovirus-A (PSV-A), PSV-B, PSV-C (new virus species infecting pineapple germplasm), and PSV-D (new virus species mined from an HTS dataset from China); **(c)** Phylogeny of the RT-RH1 nucleotide region of virus members infecting pineapple and classified within *Badnavirus* species with their homologs: pineapple bacilliform CO virus (PBcoV), and pineapple bacilliform ER virus (PBerV). Cauliflower mosaic virus was used as an outgroup.

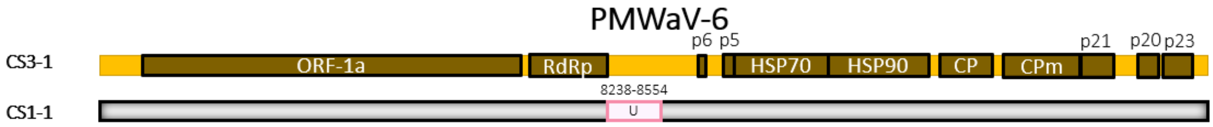

Figure S3. Map of recombination patterns and parental lineages of pineapple mealybug wilt-associated viruses-6 (PMWaV-6). Major parents are represented in light shade and minor parents in colored boxes. Events highlighted in light gray in the table were not considered for the analysis since did not provide the support for at least three out of the seven algorithms available in the RDP4 software. No recombination events were found in CS3-1 and the reference isolate of PMWaV-6 MW269512. U, unknown.

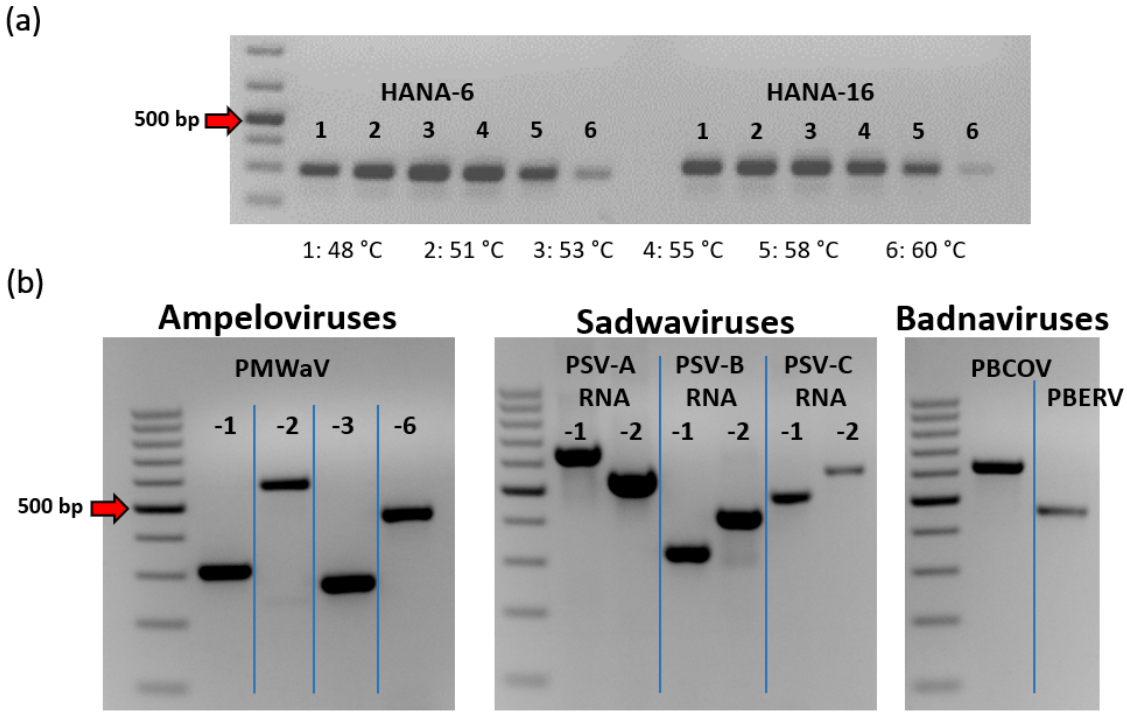

Figure S4. RT-PCR detection methods for viruses infecting pineapple: **(a)** Gradient PCR for PSV-B RNA-1 using two germplasm accessions, HANA-6 and HANA-16; **(b)** RT-PCR amplicons observed in a 1.5% agarose gel. RT-PCR products were amplified using virus-specific primer sets (Table 4.2) for ampeloviruses (PMWaV-1, -2, -3 and -6), sadwaviruses (PSV-A, -B, and -C) and badnaviruses (PBCOV and PBERV). PSV-D was originally characterized from a dataset from China mined from the transcriptome shotgun assemble (TSA) database. PSV-D was not found in any Ananas germplasm accession used in virus indexing in this study. PMWaV, pineapple mealybug wilt-associated virus; PSV, pineapple secovirus; PBCOV, pineapple bacilliform CO virus; PBERV, pineapple bacilliform ER virus

Table S1. Number of high-throughput sequencing (HTS) reads and depth of coverage values for the viral contigs retrieved from pineapple germplasm accessions.

Composite Sample (HTS)	Country of Origin	No. Raw Reads	Virus	Variant	GenBank accession	Average Length (nt)	No. Mapped Reads	Min. Coverage (No. Reads)	Max. Coverage (No. Reads)	Mean Coverage (No. reads)	Nt Identity to Reference (%)
CS1	Brazil, Colombia, Guatemala, México, Panamá, Paraguay, Puerto Rico, Trinidad, USA, Venezuela	63,982,610	PSV-A RNA1	1	OP860242	6,185	16,839	1	2,360	204.1	78.9%
				2	OP860243	6,461	16,943	1	901	196.2	78.7%
			PSV-A RNA2	1	OP860261	4,198	52,555	1	2,207	937.7	88.2%
				2	OP860262	4,134	32,208	1	1,771	583.9	86.3%
			PSV-B RNA1	1	OP860259	5,967	16,579	2	616	206.6	94.6%
				2	OP860255	5,941	13,141	1	1,048	164.6	77.0%
				3	OP860250	5,911	10,284	2	534	129.5	77.3%
				4	OP860249	5,911	10,920	1	559	137.7	77.2%
				5	OP860251	5,912	10,950	1	530	137.8	77.1%
				6	OP860254	5,802	18,898	1	601	243.4	77.0%
				7	OP860253	6,015	19,709	1	789	243.9	77.3%
				8	OP860256	5,913	14,822	1	846	186.7	76.9%
				9	OP860252	5,921	20,423	1	750	257.1	77.0%
			PSV-B RNA2	1	OP860265	3,918	11,569	1	510	219.9	93.4%
				2	OP860266	3,933	12,451	1	937	235.7	94.4%
				3	OP860267	3,153	7,576	1	2,650	147.4	71.3%
				4	OP860268	3,461	9,015	2	567	193.6	72.3%
				5	OP860269	3,823	9,162	2	666	178.1	70.7%
				6	OP860270	3,823	6,314	1	431	122.7	70.7%
				7	OP860271	3,820	5,618	1	292	109.1	70.7%
				8	OP860272	3,886	15,254	1	1,381	292.6	70.9%

CS, composite sample; **USA**, United States of America; **PSV**, pineapple secovirus.
References accessions: PSV-A: RNA1 MN809923 (6,128 bp), RNA2 MN809924 (4,161 bp); PSV-B: RNA1 OM777135 (5,956 bp), RNA2 OM777136 (3,808 bp); PMWaV-1 MN539276 (13,071 bp); PMWaV-2 MN539272 (6,259 bp); PMWaV-3 MN539274 (13,298 bp); PMWaV-6 MW269512 (17,907 bp); PBCOV GQ398110 (7,451 bp); PBERV EU377673 (1,510 bp). PSV-D sequences recovered from TSA were annotated on GenBank: RNA-1 (BK062878), and RNA-2 (BK062878)

Continue Supplementary Table 1

Composite Sample (HTS)	Country of Origin	No. Raw Reads	Virus	Variant	GenBank accession	Average Length (nt)	No. Mapped Reads	Min. Coverage (No. Reads)	Max. Coverage (No. Reads)	Mean Coverage (No. reads)	Nt Identity to Reference (%)
CS1	Brazil, Colombia, Guatemala, México, Panamá, Paraguay, Puerto Rico, Trinidad, USA, Venezuela	63,982,610	PSV-B RNA2	9	OP860273	3,709	7,339	1	364	146.5	72.5%
				10	OP860274	3,821	6,905	2	907	134.4	70.6%
				11	OP860275	3,821	9,671	2	678	188.1	71.1%
				12	OP860276	4,052	10,663	1	952	195.8	70.8%
				13	OP860277	3,871	14,670	1	1,403	282.4	70.6%
				14	OP860278	3,918	13,669	1	579	260.1	71.2%
				15	OP860279	3,898	13,863	1	575	264.5	71.2%
			PBCOV	1	OP860302	7,674	2,023	1	54	19.8	79.3%
			PMWaV-1	1	OP860291	13,064	3,215	1	123	18.4	91.8%
			PMWaV-2	1	OP860299	16,252	28,082	1	324	129.0	98.7%
			PMWaV-3	1	OP860287	13,396	4,966	1	72	27.7	95.5%
			PMWaV-6	1	OP860297	17,863	3,349	1	59	14.0	95.1%
CS2	Barbados, India, Indonesia, Jamaica, Philippines, Taiwan, USA, Vietnam	63,253,044	PSV-A RNA1	1	OP860244	6,130	47,068	2	1,385	578.8	91.0%
				2	OP860245	6,127	30,164	1	1,136	369,5	90.6%
			PSV-A RNA2	1	OP860263	4,219	31,726	1	1,892	564	89.2%
				2	OP860264	3,381	24,099	1	1,266	533	88.8%
			PSV-B RNA1	1	OP860258	5,951	14,460	2	657	181.6	91.5%
				2	OP860246	5,991	20,809	1	783	260.2	76.3%
			PSV-B RNA2	1	OP860280	3,945	31,272	1	1,114	595.2	70.2%
				2	OP860281	3,968	20,045	1	803	377.8	90.1%
			PSV-C RNA1	1	OP860260	4,474	10,158	1	260	122.9	-
			PSV-C RNA2	1	OP860285	3,869	5,694	1	202	110.2	-
			PBCOV	1	OP860303	7,932	7,470	1	334	71	65.2%

CS, composite sample; **USA**, United States of America; **PSV**, pineapple secovirus; **PMWaV**, pineapple mealybug wilt-associated virus; (d), defective. References accessions: PSV-A: RNA1 MN809923 (6,128 bp), RNA2 MN809924 (4,161 bp); PSV-B: RNA1 OM777135 (5,956 bp), RNA2 OM777136 (3,808 bp); PMWaV-1 MN539276 (13,071 bp); PMWaV-2 MN539272 (6,259 bp); PMWaV-3 MN539274 (13,298 bp); PMWaV-6 MW269512 (17,907 bp); PBCOV GQ398110 (7,451 bp); PBERV EU377673 (1,510 bp). PSV-D sequences recovered from TSA were annotated on GenBank: RNA-1 (BK062878), and RNA-2 (BK062878)

Continue Supplementary Table 1

Composite Sample (HTS)	Country of Origin	No. Raw Reads	Virus	Variant	GenBank accession	Average Length (nt)	No. Mapped Reads	Min. Coverage (No. Reads)	Max. Coverage (No. Reads)	Mean Coverage (No. reads)	Nt Identity to Reference (%)
CS2	Barbados, India, Indonesia, Jamaica, Philippines, Taiwan, USA, Vietnam	63,253,044	PMWaV-1	1	OP860292	13,093	7,987	0	231	45.7	98.1%
			PMWaV-1 (d)	2d	OP860293	10,896	6,736	0	142	46.3	84.3%
			PMWaV-2	1	OP860301	16,217	37,131	1	510	170.9	98.7%
			PMWaV-3	1	OP860289	13,353	2,348	0	64	13.1	96.9%
			PMWaV-3 (d)	2d	OP860290	7,387	1,950	1	62	19.7	91.1%
			PMWaV-6	1	OP860296	17,904	5,693	1	82	23.2	98.3%
CS3	Portugal, Samoa, South Africa, Zaire	67,710,906	PSV-B-RNA1	1	OP860257	6,092	94,442	1	4,802	110.9	79.6%
				2	OP860247	5,992	9,937	1	281	124.1	77.0%
				3	OP860248	5,996	10,388	0	278	129.7	77.0%
			PSV-B-RNA2	1	OP860282	3,911	60,677	1	2,545	1161.8	71.2%
				2	OP860283	3,936	14,625	1	703	278.1	92.9%
				3	OP860284	3,888	28,048	1	1,087	540.4	71.2%
			PBERV	1	OP860304	7,623	18,468	1	1,408	182.5	90.7%
			PMWaV-1	1	OP860294	13,260	2,722	0	56	15.4	88.4%
			PMWaV-1 (n)	2n	OP860295	13,199	4,269	0	66	24.2	86.1%
			PMWaV-2	1	OP860300	16,291	45,136	1	499	207.3	98.7%
			PMWaV-3	1	OP860288	13,248	2,722	1	55	15.3	96.4%
			PMWaV-3 (n)	2n	OP860286	12,212	4,481	0	114	27.5	74.0%
			PMWaV-6	1	OP860298	17,859	3,885	0	36	16.3	98.4%

CS, composite sample; **PSV**, pineapple secovirus; **PMWaV**, pineapple mealybug wilt-associated virus; (**n**), new variant.
References accessions: PSV-A: RNA1 MN809923 (6,128 bp), RNA2 MN809924 (4,161 bp); PSV-B: RNA1 OM777135 (5,956 bp), RNA2 OM777136 (3,808 bp); PMWaV-1 MN539276 (13,071 bp); PMWaV-2 MN539272 (6,259 bp); PMWaV-3 MN539274 (13,298 bp); PMWaV-6 MW269512 (17,907 bp); PBCOV GQ398110 (7,451 bp); PBERV EU377673 (1,510 bp). PSV-D sequences recovered from TSA were annotated on GenBank: RNA-1 (BK062878), and RNA-2 (BK062878)

Table S2. Polyprotein 1 (P1) sequence nucleotide (lower left) and amino acid (upper right) percent identity comparisons between partial sequences of molecular variants of four species belonging to pineapple secoviruses (PSVs), genus *Sadwavirus*. Sequence comparisons are based on the coding region (polyprotein P1) of RNA1. Partial genome sequences of the first sadwavirus reported in pineapple, PSV-A (MN809923) were obtained from GenBank. PSV-D sequences (GFDK01044199) were obtained from Transcriptome Shotgun Assembly (TSA) databases.

			AMINO ACID SEQUECNE IDENTITY (%)																					
			PSV-A						PSV-B														PSV-C	PSC-D
		Sequence ID	MN809923	CS1-1	CS1-2	CS2-1	CS2-2	MWP	CS1-1	CS1-2	CS1-3	CS1-4	CS1-5	CS1-6	CS1-7	CS1-8	CS1-9	CS2-1	CS2-2	CS3-1	CS3-2	MWP	CS2-1	GFDK01044199
NUCLEOTIDE SEQUENCE IDENTITY (%)	PSV-A	MN809923		92.4	92.4	97.6	97.3	97.6	30.5	30.4	30.4	30.3	30.3	30.4	30.3	30.6	30.3	30.5	30.2	30.2	30.7	32.3	29.3	29.4
		CS1-1	80.2		99.9	93.7	92.6	92.7	30.6	30.7	30.7	30.7	30.7	30.7	30.6	30.9	30.5	30.5	30.4	30.5	31.0	32.5	29.4	29.4
		CS1-2	80.2	98.7		93.7	92.6	92.7	30.6	30.7	30.7	30.7	30.7	30.7	30.6	30.9	30.5	30.5	30.4	30.5	31.0	32.5	29.4	29.5
		CS2-1	91.1	80.8	80.9		98.8	98.2	32.3	32.2	32.2	32.1	32.1	32.2	32.1	32.3	32.1	32.3	31.8	31.8	32.4	34.6	31.1	31.4
		CS2-2	90.8	80.2	80.4	95.2		97.9	30.5	30.4	30.4	30.4	30.4	30.4	30.4	30.6	30.3	30.5	30.1	30.2	30.8	32.3	29.2	29.4
		MWP	90.5	80.2	80.4	92.2	91.1		30.7	30.6	30.5	30.5	30.5	30.5	30.5	30.8	30.4	30.8	30.2	30.4	30.8	32.5	29.4	29.5
	PSV-B	CS1-1	43.5	42.9	42.8	44.5	43.7	43.8		87.9	88.0	87.9	88.0	88.2	88.2	88.2	88.3	98.6	88.9	91.5	88.1	99.3	37.1	37.4
		CS1-2	43.8	44.2	44.3	45.0	44.3	44.0	76.7		98.9	98.8	98.7	98.4	96.8	98.7	97.4	88.1	88.3	87.6	97.4	90.6	36.8	37.9
		CS1-3	43.7	44.1	44.1	45.1	44.6	44.0	77.0	93.5		99.6	99.7	99.1	97.3	98.1	96.6	88.1	88.2	87.4	97.2	90.5	36.7	37.8
		CS1-4	43.7	44.0	44.0	45.0	44.5	44.0	77.1	93.3	98.5		99.7	98.8	97.5	97.9	96.8	87.9	88.3	87.4	97.2	90.5	36.8	37.7
		CS1-5	43.7	44.1	44.1	45.1	44.7	44.1	76.8	93.7	99.2	98.3		98.9	97.4	97.9	96.8	88.1	88.3	87.5	97.3	90.5	36.7	37.8
		CS1-6	43.9	43.9	44.0	44.9	44.5	44.0	76.7	90.3	94.6	94.2	94.4		98.2	97.5	97.5	88.2	88.4	87.6	96.9	90.6	36.8	37.9
		CS1-7	43.9	43.6	43.6	44.8	44.4	43.9	76.5	87.9	91.7	91.8	91.6	96.7		95.7	99.3	88.3	88.4	87.5	96.2	90.6	37.0	38.1
		CS1-8	44.1	44.2	44.0	44.9	44.2	44.0	76.8	94.9	91.3	91.9	90.5	87.9	85.1		96.2	88.2	88.6	88.0	97.6	90.6	37.0	38.2
		CS1-9	44.1	43.9	44.0	45.0	44.3	44.1	76.2	92.3	87.4	87.5	87.3	92.4	95.7	88.5		88.4	88.4	87.7	96.3	90.8	37.1	38.2
		CS2-1	43.4	43.1	43.0	44.2	43.5	43.5	91.4	76.6	76.9	76.8	76.9	76.4	76.8	76.8	76.5		88.7	91.7	88.0	98.7	37.2	37.8
		CS2-2	43.9	43.9	43.9	44.8	44.0	44.3	76.7	76.3	76.3	76.4	76.2	76.1	76.0	76.8	76.1	76.8		89.1	88.5	90.4	37.0	37.3
		CS3-1	43.3	43.6	43.7	44.6	43.9	43.8	79.6	76.2	75.7	75.7	75.8	75.5	75.6	76.5	75.9	79.2	76.6		87.7	92.9	36.5	37.3
		CS3-2	43.8	43.8	43.8	44.7	44.2	44.2	77.0	90.1	88.0	88.1	88.0	86.1	85.3	91.2	87.3	76.9	76.6	76.6		90.4	37.0	38.3
		MWP	44.9	44.5	44.4	46.4	45.2	45.2	94.7	78.0	78.1	78.2	78.0	77.9	78.1	78.0	77.9	91.7	77.3	80.2	77.9		39.4	39.8
	PSV-C	CS2-1	38.2	38.4	38.3	39.9	38.6	38.4	48.8	48.3	48.5	48.4	48.3	48.4	48.4	48.4	48.5	49.5	48.9	48.3	48.1	50.9		74.9
	PSC-D	GFDK01044199	38.5	37.5	37.4	40.1	38.6	38.2	48.5	48.8	48.8	48.8	48.9	48.9	48.8	48.7	48.7	49.0	47.9	48.1	49.1	49.8	69.0	

Table S3. Polyprotein 2 (P1) sequence nucleotide (lower left) and amino acid (upper right) percent identity comparisons between partial sequences of molecular variants of the four species belonging to pineapple secoviruses (PSVs), genus *Sadwavirus*. Sequence comparisons are based on the coding region (polyprotein P2) of RNA2, Partial genome sequences of the first sadwavirus reported in pineapple, PSV-A (MN809923) were obtained from GenBank. PSV-D sequences (GFDK01044199) were obtained from Transcriptome Shotgun Assembly (TSA) databases.

			AMINO ACID SEQUENCE IDENTITY (%)																										
			MN809924	CS1-1	CS1-2	CS2-1	CS2-2	MWP	CS5-1	CS5-3	CS5-4	CS5-5	CS5-6	CS5-7	CS5-8	CS5-9	CS5-10	CS5-11	CS5-12	CS5-13	CS5-14	CS6-1	CS6-2	CS7-1	CS7-2	CS7-3	MWP	CS6-1	GFDK01043963
NUCLEOTIDE SEQUENCE IDENTITY (%)	PSV-A	MN809924		96.70	96.30	97.10	98.20	97.10	21.50	20.50	20.80	20.80	20.70	20.80	21.00	20.80	20.90	21.00	20.70	20.50	20.50	21.10	21.60	21.00	21.60	20.60	21.40	21.60	21.90
		CS1-1	88.60		97.30	96.70	98.00	97.50	21.70	20.60	20.80	20.90	20.80	20.90	21.10	20.90	21.00	21.10	20.80	20.60	20.60	21.30	21.70	21.10	21.80	20.70	21.60	22.10	22.10
		CS1-2	88.50	90.50		89.80	88.90	89.30	40.80	41.80	41.10	41.60	41.70	41.40	41.70	41.60	41.50	41.80	41.80	41.60	42.00	39.40	40.00	40.80	40.90	41.70	40.80	33.90	33.00
		CS2-1	89.30	89.00	89.80		91.70	90.80	40.40	41.50	41.00	41.30	41.50	41.10	41.30	41.40	41.40	41.30	41.60	41.50	41.80	39.40	39.80	40.60	40.40	41.30	40.20	33.40	32.40
		CS2-2	88.80	88.60	88.90	91.70		98.50	22.90	22.10	22.60	22.50	22.50	22.60	22.80	22.60	22.60	22.70	22.40	22.20	22.20	22.50	22.90	22.40	22.90	22.20	22.70	23.20	23.20
		MWP	89.20	89.60	89.30	90.80	91.00		21.50	20.50	20.70	20.80	20.70	20.80	21.00	20.80	20.90	21.00	20.70	20.50	20.50	21.10	21.50	20.80	21.50	20.60	21.30	21.70	22.00
	PSV-B	CS5-1	40.70	40.60	40.80	40.40	40.80	40.40		83.00	83.20	83.40	83.50	83.70	82.80	83.60	83.70	83.40	83.70	83.40	83.60	80.80	96.90	84.30	97.90	83.00	97.30	24.20	24.60
		CS5-3	41.20	41.50	41.80	41.50	41.90	42.00	74.20		93.90	94.90	94.00	94.30	93.30	93.80	95.00	94.70	95.50	94.90	95.10	80.90	82.60	87.00	83.10	98.30	82.40	24.10	25.10
		CS5-4	40.50	40.70	41.10	41.00	41.40	41.30	73.80	87.30		96.90	98.80	96.90	98.00	97.80	95.80	94.20	95.50	95.40	94.20	82.30	83.00	86.80	83.50	93.70	82.30	24.40	24.80
		CS5-5	40.70	41.30	41.60	41.30	41.70	41.90	73.20	88.00	91.20		97.00	95.30	96.80	97.80	96.30	96.50	95.90	94.80	95.30	82.00	82.90	87.40	83.50	94.70	82.60	24.00	24.80
		CS5-6	40.90	41.10	41.70	41.50	41.80	41.40	74.00	87.80	95.20	90.40		97.60	97.00	98.50	96.60	95.10	95.80	96.10	94.20	82.10	83.20	87.10	83.80	93.60	82.40	24.10	24.70
		CS5-7	40.70	41.00	41.40	41.10	41.40	41.40	74.00	87.20	97.20	92.90	96.80		95.30	96.20	99.00	97.50	98.20	96.80	94.90	82.20	83.20	87.70	83.70	94.30	82.50	24.10	24.90
		CS5-8	40.80	41.30	41.70	41.30	41.70	41.60	73.60	87.20	95.60	91.80	92.00	93.80		97.70	94.20	95.50	93.40	93.90	93.40	82.20	82.40	86.30	83.10	92.90	81.70	24.30	25.00
		CS5-9	40.90	41.30	41.60	41.40	41.60	41.70	73.80	87.70	94.50	95.40	92.90	96.20	94.90		95.10	95.10	94.30	94.80	95.10	82.10	83.10	87.20	83.70	93.60	82.50	24.10	24.80
		CS5-10	40.70	40.80	41.50	41.40	41.70	41.40	73.80	88.00	92.60	92.20	97.60	94.30	89.50	90.50		98.50	99.20	96.80	94.90	82.20	83.30	87.90	83.80	94.90	82.90	23.90	24.70
		CS5-11	40.70	41.30	41.80	41.30	41.80	41.80	73.80	88.40	88.90	93.00	94.10	91.20	92.40	91.00	95.80		97.90	95.30	94.90	82.00	83.00	87.70	83.40	94.80	82.50	24.00	25.00
		CS5-12	40.90	41.10	41.80	41.60	41.90	41.50	74.00	88.90	91.20	91.40	95.50	92.50	87.60	88.60	97.90	94.30		96.20	95.50	81.80	83.20	87.70	83.70	95.40	82.80	23.90	24.90
		CS5-13	41.00	41.10	41.60	41.50	42.10	41.90	74.40	88.10	91.90	89.20	92.40	91.70	88.40	88.70	92.30	88.70	91.60		98.20	81.70	82.90	87.80	83.30	94.90	82.10	24.00	24.70
		CS5-14	41.30	41.60	42.00	41.80	42.10	42.10	74.20	88.70	88.00	91.10	88.00	87.30	87.50	90.50	87.90	88.10	88.80	95.40		81.90	82.90	87.90	83.30	95.50	82.30	24.00	24.90
		CS6-1	39.20	39.50	39.40	39.40	39.80	39.20	72.50	72.90	73.20	72.90	73.00	73.10	73.50	73.50	72.60	72.90	72.70	73.10	73.60		80.80	82.80	80.80	80.80	80.10	23.70	24.40
		CS6-2	40.00	40.00	40.00	39.80	40.00	39.90	90.50	73.40	73.10	72.90	73.30	73.20	73.40	73.20	73.20	73.30	73.30	73.10	73.50	72.40		84.20	96.90	82.50	96.20	24.20	24.70
		CS7-1	40.30	40.60	40.80	40.60	40.60	40.60	73.80	78.10	76.60	77.20	77.20	77.00	77.00	77.50	76.90	77.20	77.10	77.20	77.90	73.70	73.80		84.20	86.80	83.20	24.50	25.30
		CS7-2	40.80	40.40	40.90	40.40	40.60	40.50	93.70	74.00	73.50	73.50	73.60	73.60	73.40	73.50	73.50	73.60	74.00	73.90	74.20	72.30	89.80	73.80		83.00	97.00	24.30	24.50
		CS7-3	41.00	41.30	41.70	41.30	41.60	41.90	74.20	96.90	87.20	88.10	87.50	86.90	87.00	87.90	87.70	88.20	88.50	87.90	89.10	73.00	73.50	77.70	73.80		82.30	24.00	25.10
		MWP	40.70	40.30	40.80	40.20	40.90	40.20	93.70	73.30	72.80	72.80	72.80	72.80	73.10	73.00	72.80	73.20	73.10	72.70	73.30	72.10	90.50	73.80	93.10	73.20		24.20	24.30
	PSV-C	CS6-1	34.00	33.20	33.90	33.40	33.70	33.50	39.40	39.70	39.30	39.30	39.60	39.60	39.60	39.40	39.40	39.70	39.10	38.90	38.90	37.80	39.40	39.40	39.40	39.30	39.30		75.60
	PSV-D	GFDK0104396	32.70	32.40	33.00	32.40	33.40	32.70	39.00	39.70	39.40	39.80	39.80	39.70	39.70	39.90	39.50	39.90	39.50	39.30	39.60	37.30	38.90	38.70	38.60	39.90	38.90	68.90	
